# Implementing a Hospital Call Center Service for Mental Health in Uganda: User-Centered Design Approach

**DOI:** 10.2196/53976

**Published:** 2024-06-06

**Authors:** Johnblack K Kabukye, Rosemary Namagembe, Juliet Nakku, Vincent Kiberu, Marie Sjölinder, Susanne Nilsson, Caroline Wamala-Larsson

**Affiliations:** 1 SPIDER - The Swedish Program for ICT in Developing Regions Department of Computer and Systems Sciences Stockholm University Stockholm Sweden; 2 Uganda Cancer Institute Kampala Uganda; 3 Hutchinson Centre Research Institute of Uganda Uganda Cancer Institute Kampala Uganda; 4 Butabika National Referral and Teaching Mental Hospital Kampala Uganda; 5 School of Public Health Makerere University Kampala Uganda; 6 RISE - Research Institutes of Sweden Kista Sweden; 7 Unit for Integrated Product Development and Design Department of Machine Design KTH Royal Institute of Technology Stockholm Sweden

**Keywords:** mHealth, mobile health, digital health, digital solution, digital solutions, digital intervention, digital interventions, mental health, awareness, Uganda, Africa, African, user centred, user centered, design, qualitative, focus group, focus groups, call centre, call centres, call center, call centers, mental, experience, experiences, attitude, attitudes, opinion, perception, perceptions, perspective, perspectives, cocreated, cocreation, service, services, mobile phone

## Abstract

**Background:**

Mental health conditions are a significant public health problem globally, responsible for >8 million deaths per year. In addition, they lead to lost productivity, exacerbate physical illness, and are associated with stigma and human rights violations. Uganda, like many low- and middle-income countries, faces a massive treatment gap for mental health conditions, and numerous sociocultural challenges exacerbate the burden of mental health conditions.

**Objective:**

This study aims to describe the development and formative evaluation of a digital health intervention for improving access to mental health care in Uganda.

**Methods:**

This qualitative study used user-centered design and design science research principles. Stakeholders, including patients, caregivers, mental health care providers, and implementation experts (N=65), participated in focus group discussions in which we explored participants’ experience of mental illness and mental health care, experience with digital interventions, and opinions about a proposed digital mental health service. Data were analyzed using the Consolidated Framework for Implementation Research to derive requirements for the digital solution, which was iteratively cocreated with users and piloted.

**Results:**

Several challenges were identified, including a severe shortage of mental health facilities, unmet mental health information needs, heavy burden of caregiving, financial challenges, stigma, and negative beliefs related to mental health. Participants’ enthusiasm about digital solutions as a feasible, acceptable, and convenient method for accessing mental health services was also revealed, along with recommendations to make the service user-friendly, affordable, and available 24×7 and to ensure anonymity. A hospital call center service was developed to provide mental health information and advice in 2 languages through interactive voice response and live calls with health care professionals and peer support workers (recovering patients). In the 4 months after launch, 456 calls, from 236 unique numbers, were made to the system, of which 99 (21.7%) calls went to voicemails (out-of-office hours). Of the remaining 357 calls, 80 (22.4%) calls stopped at the interactive voice response, 231 (64.7%) calls were answered by call agents, and 22 (6.2%) calls were not answered. User feedback was positive, with callers appreciating the inclusion of peer support workers who share their recovery journeys. However, some participant recommendations (eg, adding video call options) or individualized needs (eg, prescriptions) could not be accommodated due to resource limitations or technical feasibility.

**Conclusions:**

This study demonstrates a systematic and theory-driven approach to developing contextually appropriate digital solutions for improving mental health care in Uganda and similar contexts. The positive reception of the implemented service underscores its potential impact. Future research should address the identified limitations and evaluate clinical outcomes of long-term adoption.

## Introduction

Mental health conditions are an important public health issue globally, responsible for >8 million deaths per year [1-5]. Three million people die annually from the harmful use of alcohol, and 1 person dies every 40 seconds by suicide [1,2]. An estimated 970.1 million people (12.6% of the global population) experience some form of mental health problem [4]. Mental health conditions account for 5% of the global disability-adjusted life years and 12% to 20% of years lived with disability [4,6]. People with mental health conditions, on average, die 20 years prematurely [4,6] both due to mental as well as physical illnesses because mental health conditions are a risk factor for, or can complicate, physical illnesses, including physical injury and road traffic accidents, HIV or AIDS, cardiovascular diseases, and cancer [5,7]. People with mental health conditions also experience severe human rights violations, stigma, discrimination, abuse, and generally poor socioeconomic status [5,7-9].

Unfortunately, >75% of people with mental health problems do not have access to the care they need [1-3]. This is especially true for Uganda [10,11] and similar low- and middle-income countries (LMICs) where the treatment gap for mental disorders reaches 90% [12-14]. It is estimated that the ratio of mental health workers to population is 200 times smaller in LMICs compared with the high-income countries [3]. In LMICs, mental health is underprioritized in the face of other competing public health challenges such as HIV and AIDS, tuberculosis, malaria, and maternal and child health. Uganda, for example, spends 9.8% of its gross domestic product on health care, but <1% of this goes toward mental health care [10,11]. Consequently, Uganda experiences a shortage of mental health care facilities and professionals and poor and inconsistent access to medication and related mental health services [11]. In addition, most of the health workforce is limited to urban areas, yet >80% of the population lives in rural areas, thus geographically isolated from even the limited care available. Other important challenges facing mental health in Uganda include social norms [15], beliefs (such as witchcraft), lack of awareness of mental health disorders [8,11,16], pervasive stigma, and sociopolitical conflicts [13,17]; these not only result in an increase in the incidence of mental health problems but also lead to many people with mental health problems not seeking care and going undiagnosed.

To address some of the abovementioned challenges and improve access to mental health services in Uganda, we implemented the project *digitalizing mental health care and access in Uganda*. In this project, we followed a user-centered design (UCD) and a cocreation process to set up a hospital call center service to provide mental health information and advice to patients, caregivers, and the general public. This paper aimed to describe the development and formative evaluation of this mental health call center service.

## Methods

### Ethical Considerations

Ethical approval for the research study was obtained from the Makerere University School of Public Health research ethics committee (#SPH-2021-153) and the Uganda National Council of Science and Technology (#HS1868ES). All participants provided written informed consent before participating in the study activities.

### Study Design

We conducted a qualitative case study using the principles of UCD [18-20] and design science research (DSR) [21,22]. UCD focuses on understanding and prioritizing the needs, preferences, and behavior of end users of a product throughout its development life cycle. UCD, therefore, calls for iterative and collaborative engagement of users to ensure high usability and utility of the product. DSR is a structured approach to creating and evaluating innovative solutions or artifacts, where the design process is treated as research that contributes to knowledge for improving the functional performance of artifacts. The steps involved in DSR mirror UCD and include the following: (1) identifying the problem and motivation (understanding user experiences and context of use); (2) defining the objective of the solution (specifying the requirements); (3) designing and development of (often novel) solutions using participatory or cocreation processes; and (4) demonstrating and evaluating the solutions to validate against requirements, assess usability, and long-term adoption. These steps help identify the facilitators and barriers of adoption so that they can be addressed early on in the project life cycle, allow user engagement and facilitate buy-in, ensure that the product fits the context of use and purpose, and has good usability [23,24] and clinical utility [25].

In the following sections, we describe each of the above 4 steps. Note that there was overlap and iterations over the steps as per the UCD best practice. To ease readability, we report the procedure and results from each step. Thereafter, we provide a general discussion and conclusion.

### Step 1: Understanding User Experiences and Context to Identify the Problems

#### Participants and Recruitment

The participants included adults (≥18 years), patients recovering from mental disorders, caregivers of such patients, peer support workers (PSWs), mental health care providers, and persons involved in the implementation of call centers for telecoms or other health care centers. The health care providers, patients, caregivers, and PSWs were recruited from the Butabika National Mental Referral Hospital in Kampala, Uganda, which is also the site of implementation. Sampling was purposive to include different cadres and expertise of providers (informed by the third author, who is the head of Butabika Hospital) and to represent different mental health conditions, levels of education, and socioeconomic status of patients and caregivers to get diversity of experiences and views. The investigators (JKK, JN, and VK) physically approached the health care providers at Butabika Hospital, explained the project’s purpose and the research activities involved (including participation in multiple group discussions and workshops), and obtained consent from those interested. These health care providers then reached out to patients under their care, caregivers, and PSWs; provided them with information about the study; and invited those interested for consent by the investigators, who explained the participants’ rights and voluntary nature of participation. Participants in the last stakeholder category were recruited through the network of the first author who works in the digital health field in Uganda.

#### Data Collection

We conducted semistructured focus group discussions (FGDs) in which we explored participants’ experience of mental illness and mental health care in Uganda (including unmet information and supportive care needs); experience with call center services from the commercial service sector or other digital health care services; and opinions about a proposed digital mental health service (ie, feasibility, appropriateness, expected benefits, or recommendations for successful implementation). The FGD guide is shown in Textbox 1. There was flexibility in the order of the probes to allow free flow of ideas, with additional probes for clarification added by moderators as issues of interest arose. In addition, certain issues or probes were discussed in detail, paraphrased, or left out as appropriate depending on relevance to the session participant or if such a topic had been sufficiently explored in the prior sessions.

The sessions were conducted in English and Luganda (the lingua franca in Uganda) as appropriate for the participant category. In addition, we held male-only and female-only FGD sessions for patients to reduce the possibility that some participants would overshadow others during the discussion, but other sessions were mixed to ensure rich discussions since diverse viewpoints from different participants inspire others and spur discussion. The first and fourth authors (JKK and VK) were the moderators, while the second author (RN) was a notetaker. The sessions were audio recorded and later transcribed by the second author, who also translated the sessions in Luganda into English for analysis. The FGDs took place in November 2021.

**Figure 1:**
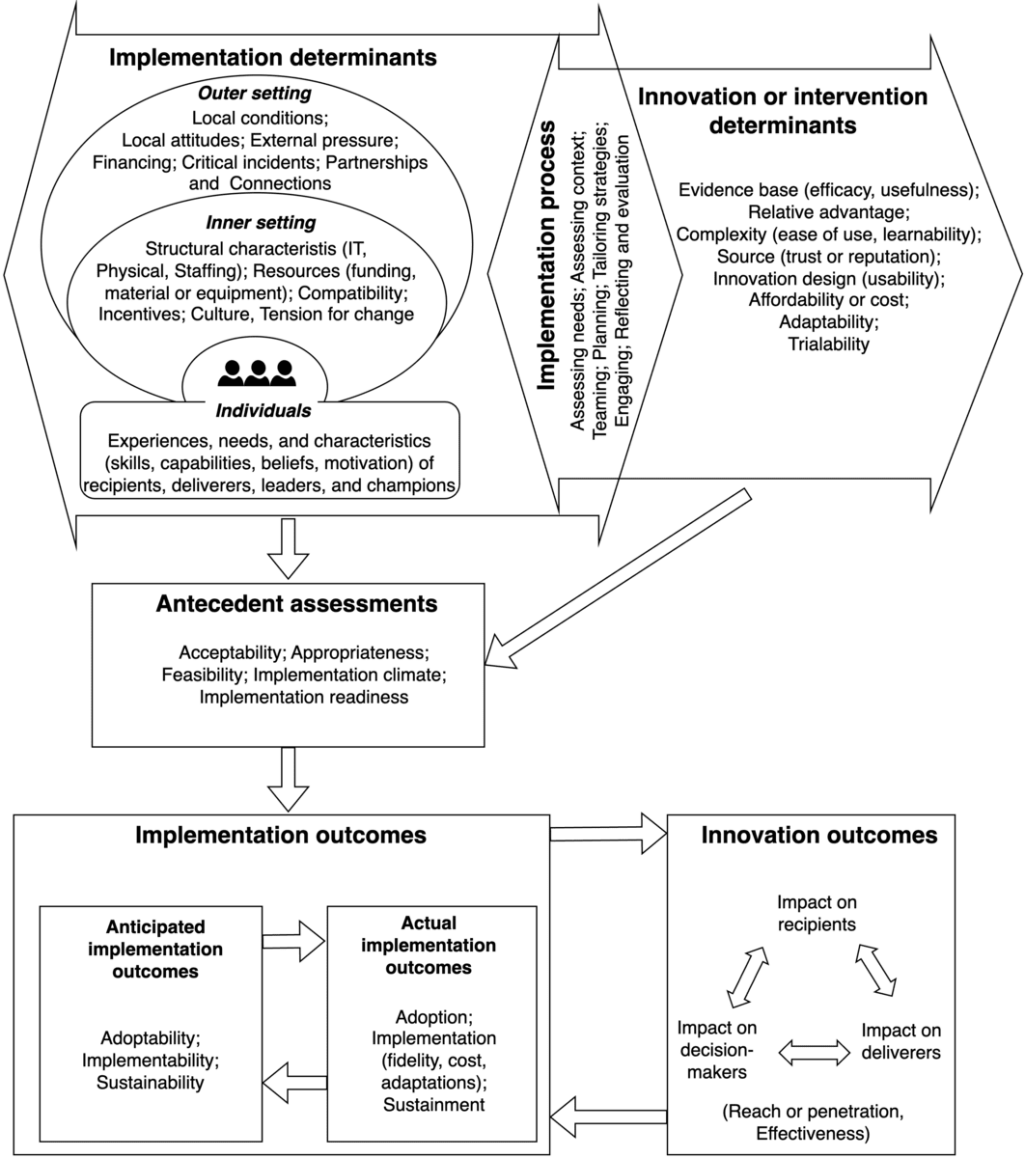
The Consolidated Framework for Implementation Research (CFIR) used in this study [26,29,32].

We drew on the Consolidated Framework for Implementation Research (CFIR) [26] to inform data collection (and analysis; see the *Data Analysis* section). The CFIR is a *metatheoretical framework* developed by consolidating several implementation science theories into one comprehensive taxonomy of clearly defined, nonoverlapping constructs related to disseminating and implementing evidence-based interventions. These constructs fall into five domains: (1) the individuals affected or involved in the implementation, (2) the innovation (intervention), (3) the inner setting (organization) where the innovation is implemented, (4) the outer setting (wider societal context), and (5) the process of implementation. The CFIR is one of the most widely used theoretical frameworks to identify implementation barriers and facilitators (ie, a determinant framework) [27-31]. Following a literature review and feedback from researchers who have used the CFIR [29], a recent update, dubbed “CFIR 2.0,” has been made, in addition to a CFIR outcomes addendum [32]. These updates have provided further clarification between constructs, including, for example, a distinction between “implementation determinants,” which relate to the context, versus “innovation determinants,” which relate to the characteristics of the innovation (eg, ease of use, relative advantage, cost, and efficacy of a technology). The implementation and innovation determinants inform the implementation process (needs assessment, user engagement, tailoring to user needs, incentives, marketing, etc) and moderate the anticipated and actual implementation outcomes through the antecedent assessments (tension or readiness for change, feasibility, acceptability, appropriateness, etc). The updates to the CFIR make it also useful for informing the design, implementation, and evaluation of innovations (ie, a process and evaluation framework) [30,31]. Figure 1 shows an adaptation of the CFIR and its recent updates as used in this study [26,29,32].

**Focus group discussion guide.**

**Topic and questions or probes**

- Participants’ understanding of mental health and mental problems
  What is mental health, and what is mental health illness?
  Do you know any forms of mental illness? What are the signs and symptoms?
  What do you do with a person who has mental problems? What have you experienced? What is usually done, and what should be the correct thing to do?
  Where can one get treatment? Probe about alternative healers, witchcraft, religious healers, etc
  How are mental health or mental illnesses viewed in your community? Probe about stigma, myths, fear, and marginalization
- Mental health information, psychoeducation, and psychosocial support
  What information about mental health problems or mental health care do you wish you knew early on in your mental illness journey?
  What issues or topics do you think are the most important to address now? Are there any topics or issues that you still need information about? Give examples.
  How or where do you get information about mental health and mental illnesses? Which ones are the best or preferred?
  Tell us any challenges or limitations of these information sources.
  Are there any services or persons that support you to cope with mental illness or care for your relatives with mental illness? Tell us more about these.
- Telemental health services
  Tell us about your experience with interactive voice response (IVR) system or call centers: which industry or business? Any challenges and advantages? (moderator to explain IVR if participants do not know and can use the examples of telecoms or bank customer care lines to explain)
  What are your thoughts on using such IVR systems for mental health information and care (telemental service)? Probes any experience of telemental health services, anticipated benefits, limitations, considerations on how to make it work, concerns about timing, phone ownership and access, privacy, etc. Probe for details and examples.
  What are the likely barriers or facilitators for such a service?
  Any thoughts about staffing and the role of peer support workers (PSWs)? Probe about acceptability to patients, benefit to PSWs, any anticipated challenges, and how to mitigate them.
  Any other thoughts about using technology in mental health care?

#### Data Analysis

A directed (deductive) content analysis approach [33] was used. We began with a rapid qualitative analysis [34-36] of the FGDs in order to quickly identify the requirements and other insights needed to inform initial iterations of system development (see *Step 2: Specifying the Requirements of the System* section). Rapid qualitative analysis is aimed at getting actionable and targeted insights in a timely manner and is suitable for studies such as this one, where there is a need to refine and adapt an intervention or program, as opposed to developing new theories. A deductive approach is taken, using existing theories or frameworks (in our case, the CFIR) to summarize the qualitative data into, for example, intervention characteristics or barriers and facilitators. In rapid qualitative research, data collection and analysis occur concurrently and iteratively, with findings from one phase informing the next iteration. The analysis is done not on the transcripts but on the summaries or notes taken during the FGDs or the audio recordings. In addition, multiple data collection methods are used to triangulate findings (eg, FGDs, field observations, debriefing, and reflections by the research team or other stakeholders and literature review). In our study, the rapid analysis was done by the first 4 authors (JKK, RN, JN, and VK) and involved note-taking during FGDs and discussion and summarization of insights after each session. When necessary, recordings were listened to by researchers who, for example, was not present in the session before they contributed to the analysis or for validating the summaries. We summarized the findings into an initial list of mental health information topics to be covered by our system and design considerations (system requirements) based on the experiences and expectations of users, as well as contextual constraints.

Later, a traditional qualitative analysis was done [34-36]. The first and second authors (JKK and RN) independently read 1 of the 7 transcripts and extracted meaningful units or statements and coded them into themes related to the research objectives and the CFIR. They then met to discuss and refine the coding before independently coding the remaining transcripts. Three more meetings were held to compare and refine the coding, after which the findings were shared with all the authors for discussion and interpretation. We focused on saliency [37] rather than frequency of issues and codes, such that even if an issue was mentioned once or by 1 participant category, we coded it as long as it related to the research question and CFIR constructs. As such, we did not count or rank the codes and themes. Basic Office software (Microsoft Corp) was used for coding and summarizing the qualitative data.

## Results

### Participants and FGD Sessions

We conducted 7 FGDs, each with 8-10 participants, for a total of 65 participants. The participants were fairly balanced by sex (female participants: 35/65, 54%; male participants: 30/65, 46%), and their ages ranged from 21 to 64 years, with a median of 40, IQR 12 years). Each session lasted approximately 1.5 hours. Table 1 shows the details of the FGD sessions.

**Table 1:** Details of focus group discussion (FGD) sessions (N=65).

FGD session	Stakeholder category	Participants, n (%)	Sex	Language	Notes
1	Patients	10 (15)	Female	Luganda	Sessions in Luganda (the most commonly spoken local language) and separated males and females to ensure participants speak freely and not overshadowed by opposite sex. Diagnoses represented included bipolar affective disorder, schizophrenia, and psychosis.
2	Patients	8 (12)	Male	Luganda	—^a^
3	Caregivers	9 (14)	Mixed	English	Separate sessions in English and in Luganda to get opinions from participants of different education status (English is learned in school in Uganda and is proxy for education and socioeconomic status). Diagnoses represented included bipolar affective disorder, schizophrenia, psychosis, epilepsy, and alcohol and substance use disorder.
4	Caregivers	10 (15)	Mixed	Luganda	—
5	Health care providers	10 (15)	Mixed	English	Staff of Butabika Hospital involved in care for patients and community outreaches, including psychiatrists, psychologists, psychiatric nurses, and psychiatric clinical officers.
6	PSWs^b^	10 (15)	Mixed	English	Volunteers with lived experience of mental illness. They work with Butabika Hospital to share their personal experience and support and educate other patients. They receive small stipends from the hospital, patients they help, or projects and grants to facilitate their work. Diagnoses represented included bipolar affective disorder, schizophrenia, and psychosis.
7	Implementers	8 (12)	Mixed	English	Customer care for telecoms, developers of IVR^c^ systems, and implementers of hospital call centers in HIV or AIDS and cancer, private telemedicine company (general care), and mental health NGOs^d^.

^a^Not applicable.

^b^PSW: peer support worker.

^c^IVR: interactive voice response.

^d^NGO: nongovernmental organization.

### Findings From the FGDs

Multimedia Appendix 1 shows the qualitative findings, including the CFIR domains, constructs, themes, and their explanation. Multimedia Appendix 2 contains illustrative quotes.

Overall, 39 themes emerged across 20 CFIR constructs in all the 5 domains and the antecedent assessments. The themes recurred across the participant groups, supporting their validity. The themes under the *individuals domain* highlighted several challenges that people with mental health conditions in Uganda face, including the limited number of mental health care facilities, long distances to care, lack of mental health information, stigma against patients with mental health problems and their families, financial challenges, and unmet psychosocial needs. The themes also covered contextual issues that explain these challenges. These included issues about the nature of mental illness (chronic and with a high burden of caregiving); organizational issues (*inner setting*), such as understaffing of mental health facilities and frequent medication stockouts; and societal issues (*outer setting*), such as beliefs and cultural norms (eg, belief in witchcraft), which influence how people understand mental health problems and how they seek care. Themes under the domain *innovation determinants* covered participants’ perception or expected benefit from the proposed mental health call center service, including affordability; familiarity with similar services and the technology (ubiquitous access to mobile phones); convenience; time and cost saving; and anonymity offered by telephone services, which protect users from the stigma. Finally, themes in the *implementation process* domain encompassed mostly participants’ recommendations or strategies for successful implementation, such as linkage with other stakeholders involved in mental health care, marketing of the service (sensitization), training and supervision of staff for quality control, and the need to maintain the human touch rather than attempting to digitalize or automate mental health care delivery. These findings suggested the feasibility, acceptability, and appropriateness of the proposed solution (*antecedent assessments*).

There were also several insights or implicit findings not mentioned by the FGD participants but inferred from observations and the research team’s understanding of the context. These are relevant for the implementation and can be mapped to CFIR constructs. For example, there has been an increase in the adoption of telemedicine in Uganda, especially following the COVID-19 pandemic, which has given credibility to such innovations and can explain the general enthusiasm shown by the participants (CFIR construct “evidence base” in *innovation determinants*). In fact, the participants in the implementers’ category were themselves involved in implementing call centers for HIV or AIDS, private telemedicine clinics, and mental health NGOs and were aware of the growing scientific evidence globally that supports digital health. The COVID-19 pandemic is also an example of “critical incidents” that can disrupt (or encourage) implementation and delivery of innovations (*outer setting*) according to CFIR 2.0. Other issues included the external project grant (construct: “Financing”), Uganda government’s positive digital transformation strategies and policies (construct: “External pressure”), and the position of Butabika Hospital as a national referral that is supposed to be exemplary (construct: “Performance measurement pressure”).

### Step 2: Specifying the Requirements of the System

#### Procedure and Team

Requirements were specified based on the understanding of the users’ needs, challenges, and contextual constraints from the FGDs. The development team consisted of the first 3 authors (a physician and digital health expert, a research nurse, and a senior consultant psychiatrist, respectively), as well as a psychologist, a psychiatric nurse, and an IT professional specializing in telephone systems. The first 2 authors and the IT professional have previously worked together to set up a similar system at the Uganda Cancer Institute [38] from which they also drew insights. The team held 8 web-based meetings from December 2021 to March 2022 to iteratively discuss the system features, content (mental health information), and setup considerations. We started with the initial list from the rapid qualitative analysis (see the *Data Analysis* section), which we refined to remove conflicting requirements or those that are not feasible due to available resources (eg, video telemedicine). We also agreed on the priority features and mental health information topics.

#### Results

Multimedia Appendix 3 lists the high-level requirements and how they were addressed in the system design and implementation. The key of these requirements is that the system or the intervention provides correct mental health information and psychosocial support in a culturally sensitive and nonstigmatizing manner and in multiple languages. In addition, the system should be easy to use (navigate), accessible 24×7, and affordable (free) to users; there should be no long queues; and it should fit within the workflow of the staff and not increase their workload. Finally, it should ensure privacy and confidentiality to users’ information, and risks of harm to users should be minimized through quality control measures, training, and professionalism of staff.

### Step 3: Design and Development of the System

#### Procedure and Team

**Figure 2:**
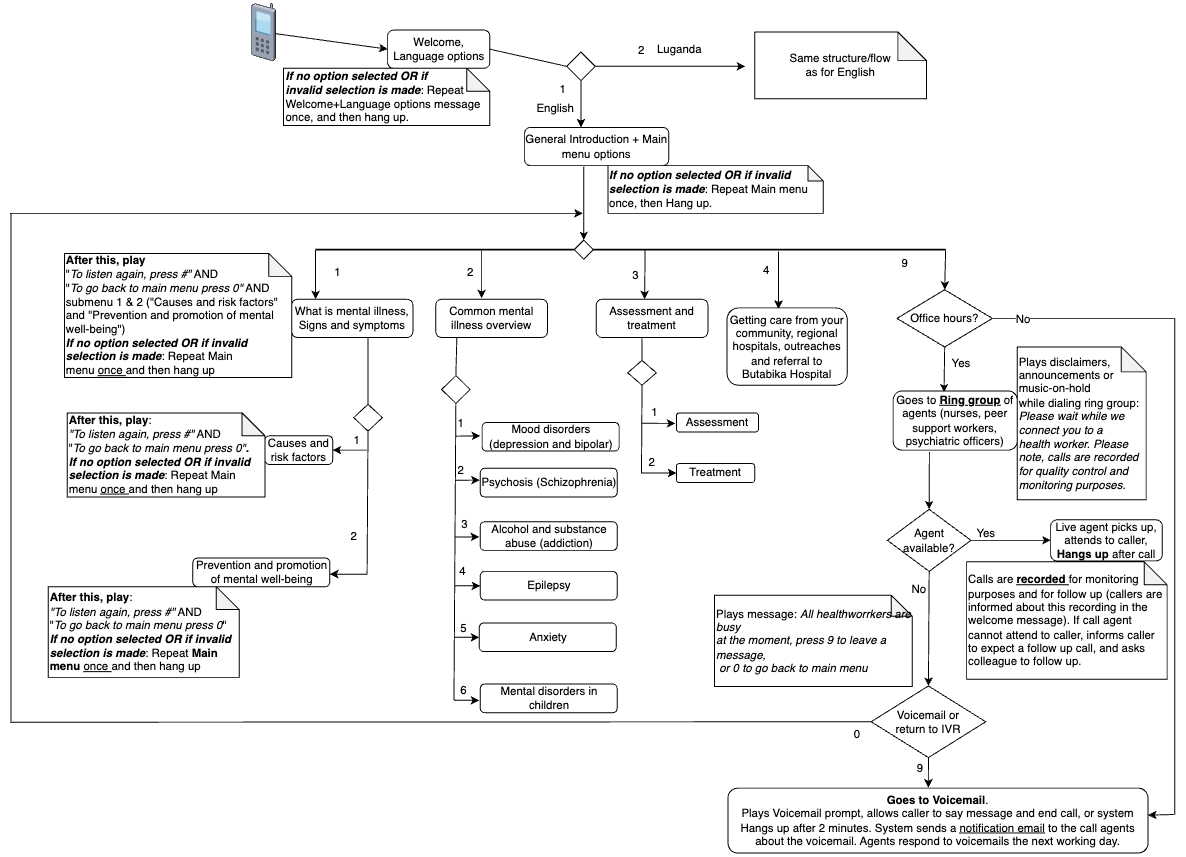
Mental health information topics and how they are accessed in the interactive voice response (IVR).

We designed and developed a telephone system for providing mental health information and advice to callers as per the requirements (Multimedia Appendix 3). The system consists of 3 complimentary components or features: an interactive voice response (IVR), live calls, and voicemails. The IVR is the first component that users interact with, and since it is automated, it is available 24×7. It contains mental health information in audio format in English and Luganda. Callers get navigation instructions and choose from a menu of topics in a self-service manner by pressing the corresponding keys on their phones (eg, *“Thank you for calling Butabika Hospital. Please choose your preferred language. For English, press 1, Bw’oba oyagala kuwuliriza mu Luganda, nyiga 2”*). Figure 2 shows the IVR flow and the topics covered.

From the IVR, callers can choose to speak directly (live call) with an agent, for example, to seek more clarification on information in the IVR or ask for information that is not covered by the IVR. If it is during office hours, the system connects the caller to the agent. We had a total of 8 agents comprising 2 PSWs, 1 psychologist, 2 psychiatric clinical officers, and 3 psychiatric nurses. The staff do not sit in a physical call center; rather, they are accessible via dedicated mobile phones. All their phones are dialed concurrently (“ring all” strategy), and whoever picks first responds to the caller. Outside working hours, callers are instructed by the system to leave a voicemail, and the call agents returns the calls the next day; this is only possible from a softphone on a computer within the hospital since the caller’s number is hidden on the agents’ mobile phones for privacy reasons. All agents were encouraged to respond to the calls immediately, and a schedule was created for responding to voicemails. The psychologist provided supervision to the agents and handled any difficult cases, which the agents were encouraged to report or escalate whenever necessary.

Development of the system began by developing the IVR content (mental health messages and navigation instructions), which was done concurrently with the requirements specification process described in *Step 2: Specifying the Requirements of the System* section. The team iteratively wrote the script for mental health messages based on their clinical expertise, reviewed the Luganda translation, and discussed the IVR menu options and caller-system interaction based on the requirements and insights from prior work. We limited the IVR options to a manageable number and organized the information in a logical order, that is, from general information (overview of mental illnesses) to specific information (eg, individual illness such as anxiety or depression). Attention was paid to ease of language (eg, description of concepts or illnesses in addition to naming them and reduction in use of medical jargon); tone (calm, empathic, and nonjudgmental); and cultural appropriateness (eg, acknowledging the role of faith and alternative medicine). The developed content was recorded in a professional audio recording studio and deployed in private branch exchange (PBX) software by the IT professional and the first author.

#### Results

The telephone system was implemented using Issabel (Issabel LLC), an open-source PBX software based on Asterisk (Sangoma Technologies Corp). It was deployed on a simple server (Intel Core i5 2.6 GHz, 8 GB RAM, 1-TB Hard disk) at Butabika Hospital and connected to a local mobile telecom provider via session initiation protocol with 12 trunks. The calls to the system are reverse billed and therefore are free to the callers.

In sum, we developed a total of 22 messages, 14 (64%) of which were on mental health or other practical information needs elicited from the participants, that is, overview of what mental illnesses are; the causes, signs, and symptoms; the common mental illnesses in Uganda; assessment and management of mental illness; and how to navigate the health care system. The remaining 8 (36%) messages contained navigation instructions or feedback to user (welcome message, language selection, disclaimer, warning in case of emergency, the different menu options, invalid selection, message replay, returning to main menu, and voicemail instructions). The messages were then translated to Luganda for a total of 44 messages. Figure 2 shows the topics addressed by the IVR messages (without some of the navigation messages).

### Step 4: Demonstration and Evaluation

#### Procedure and Participants

Following deployment, we held a 1-day workshop with the PSWs, nurses, and psychiatric clinical officers (n=10) who had participated in the FGDs to test the system, get feedback about the IVR content, and identify and correct any system malfunctions or errors (eg, if there were language mix up or a wrong response for a particular IVR option chosen by the caller).

We held a second workshop to train the call agents on workflows, software system, and phone etiquettes and how to communicate with persons with mental health problems. We also discussed operational issues, for example, definition of office hours when live calls should be allowed, and schedules for returning voicemails and evaluation survey.

The system was advertised via the hospital website and social media channels, posters in the hospital, and personal contacts of the staff and participants. After go-live, we continued to supervise the call agents and held regular review meetings in which we listened to recorded calls and critiqued the conversations, offered support to the call agents (especially the PSW) in case of difficult calls, and collected feedback on usability and user perception of utility of the service.

#### Results

No major problems were found during the testing workshops, but participants reported that the workshops helped them better understand the service from practically trying it out. They showed enthusiasm for their roles as call agents and became ambassadors who advertised the service to patients and their social networks. Schedules were also drawn for returning calls and office hours defined, which were then programmed into the PBX, sending calls outside these hours to voicemail.

The system went live in August 2021. Detailed results from a survey of the callers and analysis of the use patterns will be reported in a separate study (under preparation), but here we summarize the observations from the first 4 months of operation.

From August to December 2022, a total of 456 calls, from 236 unique numbers (average of 4 calls per day), were made to the system, that is, reaching at least the IVR (automated) component. Of these, 99 (21.7%) calls were made during out-of-office hours for the call agents, so they went to voicemail and were called back within the following days. Of the remaining 357 calls made during office hours, 80 (22.4%) calls stopped at the IVR, while 231 (64.7%) proceeded to speak to a live agent (note that the percentages do not add up to 100% because some callers made multiple calls using the IVR or leaving a voicemail and later called and spoke to a live agent). Furthermore, the 22 (6.2%) calls were never answered by the call agents. On average, live calls were answered within 11 (SD 7) seconds, and their average length was 3.5 (SD 2.8) minutes.

Callers came from all parts of the country (as far as 8 hours by road from Butabika Hospital), although the majority were from the central region (within a 1-hour distance from Butabika Hospital). They included caregivers seeking advice about relatives who were showing symptoms of mental illnesses or those already undergoing care; mental health patients who were relatively stable and were seeking advice about medication or return dates; and others such as clinicians from other health facilities, journalists, and government officials who wanted more information about the call center system or the mental health care services offered at Butabika Hospital. Calls about patients who had “escaped” from the hospital were also common, often made by concerned community members near the hospital who come across a person with mental illness wandering in the community. Generally, the service has been received positively. Callers were especially happy with the PSWs who shared their personal journeys with mental illnesses and recovery, and this encouraged them to overcome the stigma and negativity that they had about mental health care services. The PSWs also reported positive experiences, stating that working as call agents and helping others gave them a sense of purpose and brought order and calmness.

A key challenge was callers who required specific and individualized information that the call agents did not have at hand and could not be prerecorded in the IVR. Such information included requests for prescriptions, questions on stocks of certain medications, availability and cost of certain tests and procedures, or about the condition of a relative who was admitted in the hospital.

### Reflexivity

The members of the research team who were involved in data collection and analysis (FGDs, workshops, and analysis meetings) are intimately familiar with the local context and understand participants’ realities (including participants’ access and use of mobile phones and the internet) since they come from the same region of the country and speak the local language (Luganda). This made it easier to communicate with the participants (even for sessions that were held in Luganda) and to understand and relate to the ideas or issues they raised. To reduce potential undue coercion, the clinicians involved in the care of the participants (patients and PSWs) did not participate in the FGDs sessions but participated in data analysis and interpretation. These clinicians were especially important in ensuring that the rest of the research team members were aware of assumptions and potential prejudices, for example, with regard to beliefs in witchcraft as a cause for mental illnesses or in faith healing, common among those with low education status. Clinicians working in mental health care in this context frequently encounter such beliefs and appreciate the importance of respecting them, which was also useful for informing how we crafted the mental health messages in the system. Moreover, 3 of the research team members were from a different high-income country and brought in different perspectives, which helped us question our interpretations and assumptions.

## Discussion

### Principal Findings

This paper describes the development and implementation of a digital health intervention aimed at improving mental health care in Uganda. Using principles of UCD [18-20] and DSR [21,22], we systematically engaged stakeholders, collected data on target users’ experiences of mental health care, their opinions and recommendations about the proposed mental health telephone service, and contextual issues that could influence implementation. We used the CFIR, an established implementation science meta-framework [26-31], to collect and analyze these data and derive system requirements and then iteratively cocreated and tested the system.

We identified several challenges faced by patients with mental health problems and their caregivers in Uganda and peculiarities about the organization and the wider societal context, which supported the proposed innovation. These challenges included the severe shortage of mental health workers and services, lack of awareness, negative beliefs and norms, stigma, huge burden of caregiving, and financial challenges. At the same time, there is a general trend toward digitalization of health care to improve patient experience and efficiency of health care, and participants were enthusiastic about our proposed call center because they were familiar with the technology and considered it as a feasible, affordable, convenient, and efficient way to get mental health services without being stigmatized. The participants also gave several recommendations on how to successfully implement the intervention, for example, by making calls toll free, ensuring 24×7 availability, providing mental health information in multiple languages, using technologies or channels that are appropriate to the context (telephone calls and IVR), sufficient staffing to reducing call waiting times, sensitizing people about the service, and training and supervision of the call agents to ensure quality service. Early evaluation of the intervention shows that clients are very positive about the service, particularly with the use of PSWs (recovering patients) who share their lived experience with others.

### Comparison With Prior Work

Prior research has demonstrated the value of mobile health (mHealth) in addressing some of the health care challenges in Uganda and similar contexts elsewhere. Systematic reviews on mHealth in general [39-45] or on specific clinical domains such as HIV or AIDS [45-47] and palliative care [48] have highlighted the improvement of health care coordination and communication between patients and health care providers, patient adherence to treatment and reduction of loss to follow-up, patient engagement and self-care, facilitation of community-based care, and improvement of access to care for rural or geographically isolated populations. Advantages such as ubiquity of mobile technology, affordability and acceptability by patients and health workers, interactivity and personalization, and saving of time and cost of traveling to health facilities have been cited. Examples of prior studies on mHealth in Uganda include use of IVR, SMS text messages, and phone calls to support the management of HIV or AIDS [49,50] and tuberculosis [51]; use of IVR to address barriers to fistula care in Uganda [52]; SMS text messages for stroke rehabilitation [53]; and IVR for provision of cancer awareness and advice [38]. There is also a commercial digital health company that has operated different mHealth services in Uganda for approximately 10 years [54]. Unfortunately, the use of mHealth in mental health in Uganda and Africa in general is limited [8,55,56]. This is likely due to the general underfunding of mental health care services [10-14]. Available research on mHealth in mental health is mostly from developed countries [57-61], with many interventions using the internet and smartphone apps, which might not be accessible or affordable in Uganda or other LMICs. Interventions that use basic phone features such as SMS text messages, IVR, and voice calls are more appropriate in LMICs as they overcome infrastructural limitations. Such interventions are also relevant for low-income and migrant communities in developed countries since these populations face low digital health literacy and language barriers [62-65].

In the previous project led by the first author for the provision of cancer information [38], similar findings in terms of challenges faced by patients, requirements and recommendations for the system, and generally positive reception after implementation were reported. The cancer awareness system mainly used the IVR feature with prerecorded information, with the option to speak to a live agent added as an emergency due to the COVID-19 pandemic. The agents were health care workers (nurses and physicians) who, due to travel restrictions, had been free to handle phone calls. While callers appreciated this feature, it is otherwise not possible given the limited number of health workers. In this study, PSWs helped to address the shortage of health care professionals. A large multinational research study from Uganda and elsewhere has demonstrated the positive benefits of using PSWs, both for their own recovery and for the health care system [66-68]. Our study builds onto this prior work to innovatively and efficiently put this underused resource to use through digital health.

### Strengths and Limitations of the Study

A strength of this study is the strong theoretical underpinning. Implementation studies have been faulted in the past for not being theory driven, which undermines the adoption of digital technologies [30]. The UCD and DSR approach used informed a systematic cocreation process of intervention development with user participation, while the CFIR allowed a comprehensive review of user, technological, and contextual issues to inform system requirements. Even so, we could not consider all the requirements or recommendations by the participants when designing the system because of resource limitations or contradictions. For example, some participants recommended adding video calling features to the system to enhance interaction and assessment of affect. Other participants had concerns about continuity of care, which indeed is difficult to achieve with the current call center system that lacks electronic medical records or mechanisms to ensure that callers are directed to agents with whom they have interacted with before. However, adding such features would make the system complex, expensive, and inaccessible to some such as those who mentioned inability to work with smartphones or had connectivity problems. Still, the insights from this comprehensive assessment can inform future incremental iterations of the system during scale-up.

### Conclusions

Participants were enthusiastic about the proposed call center because they were familiar with the technology and considered it as a feasible, affordable, convenient, and efficient way to get mental health services without being stigmatized. The system provided mental health information and linkage to health care providers and PSWs. The information in audio format made it accessible even to the people with low literacy, and the automated IVR allowed 24×7 access while reducing the pressure on the health care workforce. Translation to English and Luganda, the 2 most spoken languages in Uganda, increased reach, as did the reverse billing (no cost to the caller) and the use of basic telephone calls as the channel of access since many Ugandans still do not have affordable and reliable internet access.

### Recommendations

In this study, people with mental illness, caregivers, and health care providers deemed a telephone-based mental health care service useful and necessary to increase access to mental health information and care and reduce stigma toward people with mental health problems. This positive view needs to be harnessed to scale up the digitalization of mental health care including providing therapy and establishing it in other mental health care settings in line with the current Ugandan digitalization policy and the Third National Development Plan. This method of mental health care may be replicable and scalable in other LMICs with mental health care system and personnel challenges similar to Uganda. Further research is needed to evaluate long-term adoption, patterns of use, and impact on clinical outcomes.

## Appendix

Multimedia Appendix 1 Summary of qualitative findings coded according to the Consolidated Framework for Implementation Research.

Multimedia Appendix 2 Consolidated Framework for Implementation Research domains, constructs, themes from focus group discussions, and illustrative quotes.

Multimedia Appendix 3 Requirements and how they are addressed by the system and its setup.

## Abbreviations

CFIR: Consolidated Framework for Implementation Research
DSR: design science research
FGD: focus group discussion
IVR: interactive voice response
LMIC: low- and middle-income country
mHealth: mobile health
PBX: private branch exchange
PSW: peer support worker
UCD: user-centered design
