# Supplementary material for: Implementing a Hospital Call Center Service for Mental Health in Uganda: User-Centered Design Approach
Source: JMIR Hum Factors. 2024 Jun 6;11:e53976. doi: 10.2196/53976 (PMC11190627; doi:10.2196/53976)
Supplement: Multimedia Appendix 1 [file humanfactors_v11i1e53976_app1.docx]

**Table 3.** Summary of qualitative findings coded according to the consolidated framework for implementation research (CFIR).

| CFIR domain, CFIR construct, and  theme | | | Explanation |
| --- | --- | --- | --- |
| **Individuals** | | | |
|  | **Experiences of recipients** | | |
|  |  | Challenges accessing mental health services | There are several challenges accessing mental health care services, particularly for those who live in rural areas. Mental health care facilities are few, limited to urban areas, and often very congested with frequent medicine stockouts. |
|  |  | Burden of caregiving | Mental illnesses come with a huge burden of caregiving, which falls on family members. This leads to burnout, worry, psychological distress, and related challenges for the caregivers. |
|  |  | Stigma | Patients with mental illness and caregivers face a lot of stigmas from community and health care setting. |
|  |  | Toxic and chronic treatment | Treatment for mental illness takes a long time and has a lot of side effects. |
|  |  | Role of faith and alternative medicine | Spirituality plays a key role in mental health care, with many patients seeking care from (or taken by their families and caregivers to) religious healers before and/or while going to mental health care centers. Alternative or spiritual healers can positively contribute to holistic patient care but can also interfere or delay proper health care seeking. |
|  | **Needs of recipients** | | |
|  |  | Unmet information needs | There is limited understanding of mental illnesses, their causes, and management. Alternative explanations, especially from faith healers, lead to confusion and affect health seeking and adherence to treatment. |
|  |  | Unmet supportive care needs | Patients and caregivers have significant unmet psychological support needs due to the burden of caregiving and the nature of mental illness. |
|  | **Personal attributes** | | |
|  |  | Financial challenges | Patients and caregivers face financial hardships related to extant poverty, health care costs, and loss of work. |
| **Innovation determinants** | | | |
|  | **Affordability** | | |
|  |  | Toll free access | To minimize financial barriers, access to the system should be free of charge to the users. |
|  | **Complexity** | | |
|  |  | Familiarity with call centers | Call centers are ubiquitous, for example, in the telecommunications customer service centers and so is access to (at least basic) phones. Target recipients are familiar with the technology and similar services. |
|  | **Relative advantage** | | |
|  |  | 24×7 access | Users consider or expect the system to be accessible round the clock, which is very advantageous since patients may prefer (or need) to access the service during out-of-office hours (eg, at night). |
|  |  | Convenience, time, and cost saving | Remote access to care is convenient and saves time and costs involved in traveling. Sometimes traveling to a health facility is not possible, in which case the call center would be the only avenue to get care. |
|  |  | Efficiency gains | The call center system allows maximum use of the few available mental health care workers |
|  |  | Anonymity | Given the stigma associated with accessing mental health care services, a call center system is advantageous as it offers anonymity. |
|  | **Trust** | | |
|  |  | Privacy and confidentiality | Users need assurance of confidentiality and privacy of their information in the call center system. |
|  |  | Reliability | There were concerns about network stability to allow continuous and reliable access. |
| **Inner setting** | | | |
|  | **Structural characteristics** | | |
|  |  | Staffing (workforce) | Mental health care providers are few compared with the patients load; and patients’ conditions are complex and require a lot of time. The professionals also have other obligations such as teaching. The call center should have sufficient personnel to handle the calls to avoid congestion and long call waiting times. |
|  |  | Medication stockouts | Inadequate supply of medicines and other supplies in mental health facilities. |
|  |  | Physical infrastructure | Limited physical space for patients to be comfortable when getting care. |
|  | **Culture** | | |
|  |  | Stigma (from health care setting) | Mental health is not discussed openly as is the case with other health care issues, and some health care providers stigmatize patients with mental illnesses. |
|  |  | Beliefs | Beliefs about the cause of mental illnesses, such as witchcraft or “curse of God,” mean that users seek alternative care options as opposed to modern medicine, which can lead to underuse of the service. |
|  |  | Patient-centeredness | Call agents need to be compassionate and patient with the users of the system because they are experiencing mental illness, which can affect how they communicate. They should be prepared for difficult callers. |
| **Outer setting** | | | |
|  | **Local attitudes** | | |
|  |  | Beliefs | Beliefs about the cause of mental illnesses, such as witchcraft or curse of God, mean that users seek alternative care options as opposed to modern medicine, which can lead to underuse of the service. |
|  |  | Stigma and exclusion | Patients are stigmatized and excluded, they are not involved in decision-making for their care, and sometimes families give up on them (do not take them for proper care or incarcerate them). |
|  | **Policies and laws** | | |
|  |  | Consent for minors | Potential legal dilemma when it comes to minor who cannot consent but could access the service. |
|  |  | Inertia toward telemedicine | Health care professionals are still hesitant toward remote care. |
| **Implementation process** | | | |
|  | **Teaming** | | |
|  |  | Linkage with other stakeholders | Work with other stakeholders and services providers such as the police, faith healers, and primary health care providers who are all involved in the management of people with mental illnesses. |
|  | **Engaging** | | |
|  |  | Marketing and sensitization | There is a need to market the service so that potential beneficiaries are proactively sensitized about its existence. Similar projects have used social media or bulk SMS text messaging. |
|  | **Planning** | | |
|  |  | Contingences and escalation | Incidents or cases that cannot be managed on phone can happen and a contingence plan should be in place. |
|  |  | Training and supervision | Call agents need to be trained, supervised, and supported to provide the service, especially the peer support workers who are not professionals but rather recovering patients. |
|  |  | Scope of service | Limit scope to providing general information, triage, and directions, which is more achievable and serves majority of the recipients. |
|  | **Tailoring** | | |
|  |  | System design and features | Offer different features or options for channels of communication and languages to cater for different user profiles. |
|  |  | Human touch | Patients with mental illnesses and their caregivers need a human touch, so there should be a possibility for users of the service to interact with people. The technology should not replace but complement current services delivered by people. |
|  | **Reflecting and evaluating** | | |
|  |  | Challenges with data collection | The anonymity and remote access could be that the data collected are unreliable as it is difficult to confirm them (eg, the identity of callers). This also affects continuity of care. |
| **Antecedent assessments** | | | |
|  | **Feasibility** | | |
|  |  | Technological feasibility | Access to mobile phones is ubiquitous, so it is feasible for target recipients to access the service. However, there were some concerns about network connection problems and lack of access to electricity for some users, which would affect meaningful access and use of the service. |
|  | **Acceptability** | | |
|  |  | Familiarity with call centers | Call centers are ubiquitous, for example, in the telecommunications customer service centers, and target recipients are familiar with them and can easily use them. |
|  |  | Inertia toward telemedicine | Health care professionals are still hesitant toward remote care, |
|  | **Appropriateness** | | |
|  |  | Appropriateness of digital tools for mental health care | Nonpharmacological interventions such as psychotherapy and psychosocial interventions are an important part of mental health care, and these can be delivered via remote communication platform. However, since some nonverbal communication is lost in remote services (especially voice-based communication), the service may be inappropriate in some cases where this is necessary, for example, for diagnosis or assessing affect. Some mental illnesses or states for example, hallucinations, might also be incompatible with digital tools. |
