# Supplementary material for: Implementing a Hospital Call Center Service for Mental Health in Uganda: User-Centered Design Approach
Source: JMIR Hum Factors. 2024 Jun 6;11:e53976. doi: 10.2196/53976 (PMC11190627; doi:10.2196/53976)
Supplement: Multimedia Appendix 3 [file humanfactors_v11i1e53976_app3.docx]

Table 4. Requirements and how they are addressed by the system and/or its setup.

| Requirements | System design and setup to address the requirements |
| --- | --- |
| The system or service should provide users with correct information about mental health problems: their causes, symptoms, how they are managed, and where to get care from. | - The system consists of an IVR^a^ system with information on different topics such as overview of what mental illnesses are; the causes, signs, and symptoms; the common mental illnesses in Uganda; assessment and management of mental illness; and how to navigate the health care system (Figure 2). The call agents are also trained on this information in addition to their clinical expertise as nurses, psychiatric officers, and peer support workers. |
| The system or service should provide users with psychosocial support. | - Call agents offer counseling and empathetic advice to callers. Peer support workers share personal experiences with mental illness. |
| Information provision should be culture sensitive and nonstigmatizing. | - Call agents understand the culture (including beliefs, norms, stigma around mental illnesses, and care seeking for mental illness). They also have experience communicating with patients as clinical staff of Butabika National Mental Hospital or have personal experience as peer support workers (patients recovering from mental illnesses). Furthermore, refresher training was given at the start of the project, including a workshop by personnel from a customer service center for a telecom company, a hospital call center for HIV/AIDS support, and a private telemedicine service (described in Table 2). The training covered issues as showing empathy, giving respect, being nonjudgmental, being calm, showing interest in the caller, and listening attentively. |
| Focus should be on triage, and information should be generic advice rather than individualized clinical assessment (diagnosis) or medication prescription. | - Call agents were trained and regularly given feedback on this. |
| Staff (call agents) should act professionally, remain within the limits of their expertise, and escalate complicated cases to more experienced colleagues or encourage clients to physically go to a health care facility. | - Call agents were trained and regularly given feedback on this. One of the agents (a psychologist) and the third author (a consultant psychiatrist) were designated as contact persons for escalation. There was also an instant messaging group with all call agents and the project team to consult or report issues. |
| Service should be accessible 24×7, and callers should not wait long in the queues before being served. | - IVR allows automated access (self-service) of the mental health information and therefore available 24×7. Voicemail feature allows users to ask questions even during out-of-office hours or when staff are unavailable or busy. - Multiple SIP^b^ trunks (n=12) were procured to connect to the telecom service provider, which minimizes chances of congestion for incoming calls and allows for easy scalability if more trunks are needed. - A “ring all” strategy is used for live calls, that is, for outbound calls, the system dials all available agents at the same time increasing the likelihood that the caller is responded to without waiting. - All agents are encouraged to respond to the calls immediately, and a schedule exists for responding to voicemails. |
| The service should not increase the workload of hospital staff or interfere with their workflow. | - We created mobile extensions, that is, routing live calls to mobile phones carried by the call agents as opposed to softphone extensions on the desktop to allow flexibility for the staff to answer calls even when off site, in clinics, or in wards. They do not have to go to a designated call center room. - The “ring all” strategy also allows call agents who are busy to safely ignore the call, which is then answered by colleagues in the ring group who might not be busy at that moment. - We used peer support workers (recovering patients) to supplement the limited health care workforce, and using IVR allows automation thus reducing the burden on staff. |
| Information should be available in multiple languages. | - Information is available in English and Luganda, the most commonly spoken languages in Uganda. - Other languages are planned in the future. |
| Information should be easy to understand and navigate. | - Information was carefully curated, and the IVR was organized in such a way to ensure ease of understanding and navigation. There is minimal use of medical jargon, and descriptions of concepts or illnesses are provided in addition to naming them. Information is presented in a logical order in the IVR, that is, from general information (overview of mental illnesses) to specific (eg, individual illness such as anxiety or depression). The IVR tree manageable number of options (2-6 options), and navigation instructions are repeated at the end of each branch or in case the user enter an invalid option. |
| Various channels of communication should be used to ensure that different users have access. | - The system is accessed via basic telephone calls (no internet required), making it accessible to anyone with a mobile phone. - Access strategies include IVR, live calls, and voicemail to serve different user preferences or ensure access during out-of-office hours or when there are high caller volumes. - Other features such as SMS text messaging, USSD^c^, online access, and video calls, which were mentioned, could not be implemented in this phase of the project due to resource limitations but are planned for the future. |
| There should be no financial barriers to users when accessing the system (should be free or affordable). | - System is reverse billed, that is, phone bills paid by the receiver (hospital) and therefore toll free to the callers. |
| There should be mechanisms to ensure security, privacy, and confidentiality of callers’ information. | - Call agents and members of the implementation team received training in good clinical practice and human subject protection, and issues concerning information security, privacy, and confidentiality are regularly discussed in the team meetings. - Call agents have mobile phones dedicated to the call center service and do not use their personal phones. - The phone numbers of the callers are hidden by the system when dialing the call agents; it instead displays the SIP lead number, and the agent must physically access the dedicated call center client computer on site at the Butabika Hospital to see caller details (eg, when returning a voicemail). The client computer is password protected and kept in a locked room. - The server on which the system run and where call recordings are stored is kept in a different server room that is only accessible to IT staff. |
| There should be training and ongoing supervision to ensure smooth operation and minimize the risk of harm to callers. | - Training workshops were held before system go-live, followed by mentorship and technical support for 6 months by the first author and IT consultant. - All calls were recorded for the purpose of auditing the conversations and quality control. Regular (2 weekly) team meetings were held in which any challenges are discussed, and random calls are reviewed to provide feedback and peer learning. |

^a^IVR: interactive voice response.

^b^SIP: session initiation protocol.

^c^USSD: Unstructured Supplementary Service Data.
